# 2581. Desirability of Outcome Ranking and Response Adjusted for Antibiotic Risk (DOOR/RADAR) Post-Hoc Analysis Supports Equipoise for Antibiotic Initiation Strategies in ICU-Acquired Pneumonia

**DOI:** 10.1093/ofid/ofad500.2196

**Published:** 2023-11-27

**Authors:** Christopher A Guidry, Lynn Chollet-Hinton, Jordan Baker, Jacob C O’Dell, Robel T Beyene, Christopher M Watson, Robert G Sawyer, Steven Q Simpson, Leanne Atchison, Michael Derickson, Lindsey C Cooper, G Patton Pennington, Sheri VandenBerg, Bachar N Halimeh

**Affiliations:** University of Kansas Medical Center, Kansas City, Kansas; University of Kansas Medical Center, Kansas City, Kansas; University of Kansas Medical Center, Kansas City, Kansas; University of Kansas Medical Center, Kansas City, Kansas; Vanderbilt University Medical Center, Nashville, Tennessee; Prisma Health Midlands, Columbia, South Carolina; Western Michigan Homer Stryker M.D. School of Medicine, Kalamazoo, Michigan; University of Kansas Medical Center, Kansas City, Kansas; Vanderbilt University Medical Center, Nashville, Tennessee; Vanderbilt University Medical Center, Nashville, Tennessee; Prisma Health Midlands, Columbia, South Carolina; Tallahassee Memorial Healthcare, Tallahassee, Florida; Bronson Methodist Hospital, Kalamazoo, Michigan; Boston University Medical Center, Boston, Massachusetts

## Abstract

**Background:**

Pneumonia is the most common ICU-acquired infection and source of potential sepsis in ICU populations but can be difficult to diagnose in real-time. Despite limited data, rapid initiation of antibiotics is endorsed by society guidelines. We hypothesized that a post-hoc analysis of a recent randomized pilot study would show no difference between two antibiotic initiation strategies.

**Methods:**

The recent Trial of Antibiotic Restraint in Presumed Pneumonia (TARPP) was a pragmatic cluster-randomized pilot of antibiotic initiation strategies for intubated patients with suspected but not yet proven ICU-acquired pneumonia. Participating ICUs were cluster-randomized to either an immediate initiation protocol or a specimen-initiated protocol where a gram stain was required for initiation of antibiotics. Patients in the study were divided into one of seven mutually exclusive outcome rankings (DOOR): 1) Survival, No Pneumonia, No adverse events; 2) Survival, Pneumonia, No adverse events; 3) Survival, No Pneumonia, ventilator-free-alive days ≤14; 4) Survival, Pneumonia, ventilator-free-alive days ≤14; 5) Survival, No Pneumonia, Subsequent episode of suspected pneumonia; 6) Survival, Pneumonia, Subsequent episode of suspected pneumonia; and 7) Death. These rankings were further refined using the duration of antibiotics prescribed for pneumonia (RADAR). Analysis was performed using R and adjusted for clustering.

**Results:**

There were 186 patients enrolled in the study: 93 in each group. After applying the DOOR analysis, a randomly selected patient in the specimen-initiated group was just as likely to have a better outcome as a patient in the immediate initiation group (DOOR probability: 50.8%; 95% CI: 42.7-58.9%). After applying the RADAR analysis, the likelihood of a randomly selected patient in the specimen-initiated arm having a better outcome than a patient in the immediate arm was 52.5% (95% CI: 44.2-60.6%; p=0.31).

**Conclusion:**

We found that patients in whom antibiotics were withheld until there was objective evidence (specimen-initiated group) had similar outcome rankings to patients where antibiotics were started immediately. This confirms the findings of the TARPP pilot trial and further gives evidence for equipoise between these two treatment strategies

**Disclosures:**

**Robert G. Sawyer, MD**, AbbVie: Advisor/Consultant|Merck: Advisor/Consultant|Pfizer: Advisor/Consultant

